# Teen Pregnancy and Risk of Premature Mortality

**DOI:** 10.1001/jamanetworkopen.2024.1833

**Published:** 2024-03-14

**Authors:** Joel G. Ray, Longdi Fu, Peter C. Austin, Alison L. Park, Hilary K. Brown, Sonia M. Grandi, Ashley Vandermorris, Alexa Boblitz, Eyal Cohen

**Affiliations:** 1 Department of Medicine, St Michael’s Hospital, University of Toronto, Toronto, Ontario, Canada; 2 Department of Obstetrics and Gynaecology, St Michael’s Hospital, University of Toronto, Toronto, Ontario, Canada; 3 ICES, Toronto, Ontario, Canada; 4 Child Health Evaluative Sciences, SickKids Research Institute, Toronto, Ontario, Canada; 5 Department of Health and Society, University of Toronto Scarborough, Scarborough, Ontario, Canada; 6 Dalla Lana School of Public Health, University of Toronto, Toronto, Ontario, Canada; 7 Division of Adolescent Medicine, Department of Pediatrics, University of Toronto, Toronto, Ontario, Canada; 8 Department of Pediatrics, University of Toronto, Toronto, Ontario, Canada; 9 Edwin S. H. Leong Centre for Healthy Children, University of Toronto, Toronto, Ontario, Canada

## Abstract

**Importance:**

Unintentional injury, suicide, and homicide are leading causes of death among young females. Teen pregnancy may be a marker of adverse life experiences.

**Objective:**

To evaluate the risk of premature mortality from 12 years of age onward in association with number of teen pregnancies and age at pregnancy.

**Design, Setting, and Participants:**

This population-based cohort study was conducted among all females alive at 12 years of age from April 1, 1991, to March 31, 2021, in Ontario, Canada (the most populous province, which has universal health care and data collection). The study period ended March 31, 2022.

**Exposures:**

The main exposure was number of teen pregnancies between 12 and 19 years of age (0, 1, or ≥2). Secondary exposures included how the teen pregnancy ended (birth or miscarriage vs induced abortion) and age at first teen pregnancy.

**Main Outcomes and Measures:**

The main outcome was all-cause mortality starting at 12 years of age. Hazard ratios (HRs) were adjusted for year of birth, comorbidities at 9 to 11 years of age, and area-level education, income level, and rurality.

**Results:**

Of 2 242 929 teenagers, 163 124 (7.3%) experienced a pregnancy at a median age of 18 years (IQR, 17-19 years). Of those with a teen pregnancy, 60 037 (36.8%) ended in a birth (of which 59 485 [99.1%] were live births), and 106 135 (65.1%) ended in induced abortion. The median age at the end of follow-up was 25 years (IQR, 18-32 years) for those without a teen pregnancy and 31 years (IQR, 25-36 years) for those with a teen pregnancy. There were 6030 deaths (1.9 per 10 000 person-years [95% CI, 1.9-2.0 per 10 000 person-years]) among those without a teen pregnancy, 701 deaths (4.1 per 10 000 person-years [95% CI, 3.8-4.5 per 10 000 person-years]) among those with 1 teen pregnancy, and 345 deaths (6.1 per 10 000 person-years [95% CI, 5.5-6.8 per 10 000 person-years]) among those with 2 or more teen pregnancies; adjusted HRs (AHRs) were 1.51 (95% CI, 1.39-1.63) for those with 1 pregnancy and 2.14 (95% CI, 1.92-2.39) for those with 2 or more pregnancies. Comparing those with vs without a teen pregnancy, the AHR for premature death was 1.25 (95% CI, 1.12-1.40) from noninjury, 2.06 (95% CI, 1.75-2.43) from unintentional injury, and 2.02 (95% CI, 1.54-2.65) from intentional injury.

**Conclusions and Relevance:**

In this population-based cohort study of 2.2 million female teenagers, teen pregnancy was associated with future premature mortality. It should be assessed whether supports for female teenagers who experience a pregnancy can enhance the prevention of subsequent premature mortality in young and middle adulthood.

## Key Points

**Question:**

What is the risk of premature mortality from 12 years of age onward in association with teen pregnancy?

**Findings:**

In this population-based cohort study of 2.2 million female teenagers, the risk of premature death was 1.9 per 10 000 person-years among those without a pregnancy, 4.1 per 10 000 person-years among those with 1 pregnancy, and 6.1 per 10 000 person-years among those with 2 or more pregnancies.

**Meaning:**

This study suggests that teen pregnancy may be a readily identifiable marker for subsequent risk of premature mortality in early adulthood.

## Teaser

This population-based cohort study evaluates the risk of premature mortality from 12 years of age onward in association with number of teen pregnancies and age at pregnancy in Ontario, Canada.

## Introduction

Deaths among adolescent and young women are more common than previously realized.^1,2,3,4,5^ In the US, the leading causes of death among females aged 1 to 19 years are unintentional injury, suicide, and homicide; unintentional injury, cancer, and suicide are the leading causes among those aged 20 to 44 years.^6^ Although direct deaths during teen pregnancy and childbirth are rare^7^—predominantly from hemorrhage, hypertensive disorders, or sepsis^8^—teen pregnancy may be a marker of adverse life experiences preceding and/or during the formative teen years. For example, there is a dose-response association of exposure to adverse childhood experiences (ACEs)—such as sexual and emotional abuse, parental divorce or separation, or income decline^9^—with subsequent teen pregnancy,^10,11^ substance use,^12^ and suicide.^13^ ACEs are also associated with premature mortality.^9,14,15^

The aforementioned studies reporting on the outcomes of teen pregnancies were limited by small sample sizes, self-reported outcomes later in life, incomplete data about induced abortion, and lack of information about cause of death. Population-based data within a universal health care system, like that in Canada, can capture all teen pregnancies with minimal selection bias, including hospital live births, stillbirths, and miscarriages or ectopic pregnancies, as well as drug- and procedure-induced abortions.^16,17^ The present study evaluated the rate of premature mortality from 12 years of age onward in association with the number of teen pregnancies, including the age at and nature of the teen pregnancy (ie, birth, miscarriage, or ectopic pregnancy contrasted with induced abortion), as well as the cause of death.^4^

## Methods

This population-based cohort study was conducted among all females who were alive at 12 years of age from April 1, 1991, to March 31, 2021, residing in the province of Ontario, Canada, and thus eligible for the universal Ontario Health Insurance Plan (OHIP). The index date for cohort entry was their 12th birthday. The use of the deidentified data in this project is authorized under section 45 of Ontario’s Personal Health Information Protection Act and does not require review by a research ethics board or informed consent. This report followed the Strengthening the Reporting of Observational Studies in Epidemiology (STROBE) reporting guideline for cohort studies.

All datasets were linked using unique encoded identifiers and analyzed at ICES (eTable 1 in Supplement 1). ICES is an independent, not-for-profit organization that houses diagnostic, procedural, and sociodemographic data for Ontario residents. Data at ICES are linked deterministically and include hospitalizations, emergency department visits, census data, and births and deaths and capture induced abortions of a procedural and pharmaceutical nature^17^ (eTable 2 in Supplement 1).

### Study Exposures

The main study exposure was the number of teen pregnancies experienced between 12 and 19 completed years, categorized as 0 (the referent), 1, or 2 or more teen pregnancies. The teen pregnancy date was the recorded date of either a live birth, stillbirth, induced abortion, or miscarriage. This exposure was treated as a time-varying covariate because an individual could accumulate teen pregnancies over time.

A secondary exposure was the nature of the teen pregnancy between 12 and 19 years of age: no teen pregnancy (referent); teen pregnancy ending spontaneously in a live birth, stillbirth, miscarriage, or ectopic pregnancy; or teen pregnancy otherwise ending in an induced abortion.^16^ The justification for this approach is that a teen pregnancy ending in induced abortion has been associated with a higher future risk of nonlethal self-harm.^18^ Another secondary exposure was the age at which a female experienced her first pregnancy, namely, 12 to 15, 16 to 17, or 18 to 19 years, each relative to no teen pregnancy.

### Study Outcomes

The primary study outcome was all-cause mortality starting at 12 years of age (eTable 2 in Supplement 1). A secondary outcome was all-cause mortality starting at 20 years of age (additional analysis 1 in eTable 2 in Supplement 1). For this latter outcome, women were followed up from 20 years of age onward, with individuals who died prior to 20 years of age excluded. Mortality details were further expanded by assessing the nature of the death—noninjury related, injury related of an unintentional nature, and injury related of an intentional nature—captured by the vital statistics dataset up to December 2018 (eTable 2 in Supplement 1).

### Statistical Analysis

Baseline characteristics contrasted females with teen pregnancy vs no teen pregnancy between 12 and 19 years of age using standardized differences, with a value greater than 0.10 indicating an important difference.^19^ Unadjusted and adjusted hazard ratios (HRs) for all-cause mortality from 12 years of age (time zero) onward were generated using a Cox proportional hazards regression model, with age as the timescale (main model). A participant was censored at the end of provincial health coverage (as might occur with migration out of the province) or the end of the study period of March 31, 2022. Thus, 1 year of follow-up would be possible for a woman whose 20th birthday was on March 31, 2021, and longer for anyone whose 20th birthday was before that date. The number of teen pregnancies between 12 and 19 years of age was treated as a time-varying exposure. Hazard ratios were adjusted for each woman’s year of birth (as a continuous calendar year value [eg, 1985, 1986, 1987, etc]), number of comorbidity Aggregated Diagnosis Groups (ADGs) at 9 to 11 years of age (≤2, 3-4, 5-6, or ≥7), area-level educational attainment less than high school (when the teenager was 12 years of age), time-varying residential income quintile at 12 to 19 years of age, and time-varying rural residence at 12 to 19 years of age (eTable 1 in Supplement 1). Area-level education was for the neighborhood where the teenager resided at 12 years of age based on the 6-digit residential postal code. Additional analysis further added to the main model included time-varying Mental Health ADG No. 23 (Psychosocial: Time Limited, Minor), No. 24 (Psychosocial: Recurrent or Persistent, Stable), and No. 25 (Psychosocial: Recurrent or Persistent, Unstable) at 12 to 19 years of age.

In the separate Cox proportional hazards regression models of the nature of the pregnancy (no teen pregnancy and teen pregnancy ending spontaneously or not^16,18^) and the age at teen pregnancy (no teen pregnancy, 12-15, 16-17, or 18-19 years), the earliest teen pregnancy was chosen as the index exposure event. In the assessment of the nature of the premature death (noninjury related, unintentional injury, or intentional injury), a cause-specific hazard model was used, censoring on the competing risk of the other causes of death. Of all fatal intentional injuries among those who did or did not have a teen pregnancy, a breakdown was provided for those due to self-harm (*International Classification of Diseases, Ninth Revision* [*ICD-9*] codes E950-E959; *International Statistical Classification of Diseases and Related Health Problems, Tenth Revision, Enhanced for Canada* [*ICD-10CA*] codes X60-X84 and Y87.0) and assault (*ICD-9* codes E960-E969; *ICD-10CA* codes X85-Y09 and Y87.1).

In the assessment of all-cause mortality from 20 years of age onward (additional analysis 1), the cohort was restricted to women alive at their 20th birthday who had consistent OHIP eligibility between 12 and 19 years of age. In the Cox proportional hazards regression model, HRs were adjusted for each woman’s year of birth, number of comorbidity ADGs at 9 to 11 years of age, area-level educational attainment less than high school when the teen was 20 years of age, income quintile at 20 years, and rural residence at 20 years. Statistical significance was set at a 2-sided *P* < .05.

## Results

Of 2 242 929 females, 163 124 (7.3%) had a teen pregnancy and 2 079 805 (92.7%) did not (Table 1). Those who experienced a teen pregnancy were more likely to reside in the lowest neighborhood income quintile and in an area with less completion of a high school education. Females with a teen pregnancy had a higher proportion of self-harm history between 12 and 19 years of age than those without a teen pregnancy (8123 [5.0%] vs 30 669 [1.5%]), but no higher recorded proportion of comorbidities, including those of a physical or mental health nature. The median age at teen pregnancy was 18 years (IQR, 17-19 years), of whom 121 276 (74.3%) had 1 pregnancy and 41 848 (25.6%) had 2 or more pregnancies. Of all female teenagers who had a pregnancy, 60 037 (36.8%) ended in a birth (of which 59 485 [99.1%] were live births), 106 135 (65.1%) ended in induced abortion, and 17 945 (11.0%) ended in miscarriage or ectopic pregnancy. The median age at the end of follow-up was 25 years (IQR, 18-32 years) for those without a teen pregnancy and 31 years (IQR, 25-36 years) for those with a teen pregnancy.

**Table 1.** Description of 163 124 Females Aged 12 to 19 Years Who Had a Teen Pregnancy in Ontario, Canada, Between 1991 and 2021 and of 2 079 805 Females Who Did Not

Characteristic	No. (%)		Standardized difference^a^
	Teen pregnancy (n = 163 124)	No teen pregnancy (n = 2 079 805)	
Residential income quintile at 12 y			
1 (Lowest)	49 917 (30.6)	385 413 (18.5)	0.28
2	35 802 (21.9)	378 735 (18.2)	0.09
3	30 546 (18.7)	414 288 (19.9)	0.03
4	26 445 (16.2)	446 060 (21.4)	0.13
5 (Highest)	20 414 (12.5)	455 309 (21.9)	0.25
Rural residence at age 12 y	26 966 (16.5)	268 997 (12.9)	0.10
Area-level educational attainment less than high school at age 12 y, mean (SD), %^b^	24.7 (12.2)	20.1 (10.5)	0.40
Self-harm or overdose documented at age 12-19 y	8123 (5.0)	30 669 (1.5)	0.20
No. of comorbidity ADGs at ages 9-11 y^c^			
≤2	150 444 (92.2)	1 951 642 (93.8)	0.06
3-4	9124 (5.6)	95 716 (4.6)	0.05
5-6	2591 (1.6)	23 413 (1.1)	0.04
≥7	965 (0.6)	9034 (0.4)	0.02
Mental ADG at ages 9-11 y^d^	1762 (1.1)	15 570 (0.7)	0.03
Major physical ADG at ages 9-11 y^e^	12 391 (7.6)	154 241 (7.4)	0.01
Age at first teen pregnancy, median (IQR), y	18 (17-19)	NA	NA
No. of teen pregnancies, median (IQR)	1 (1-2)	NA	NA
Teen pregnancy at ages 12-19 y that ended in a live birth or stillbirth^f^^,^^g^	60 037 (36.8)	NA	NA
Teen pregnancy at ages 12-19 y that ended in a miscarriage or ectopic pregnancy^f^	17 945 (11.0)	NA	NA
Teen pregnancy at ages 12-19 y that ended in induced abortion^f^	106 135 (65.1)	NA	NA
Age at end of study follow-up, median (IQR), y^f^	31 (25-36)	25 (18-32)	0.65

Abbreviations: ADG, Aggregated Diagnosis Group; NA, not applicable.

^a^ A standardized difference >0.10 indicates an important difference.

^b^ Percentage of the local population aged 25 to 64 years with no high school certificate, diploma, or degree.

^c^ Aggregated Diagnosis Groups were created by the Johns Hopkins Adjusted Clinical Group system using inpatient and outpatient diagnostic information from 12 to 19 years of age.

^d^ Mental ADGs refer to ADG No. 23 (Psychosocial: Time Limited, Minor), ADG No. 24 (Psychosocial: Recurrent or Persistent, Stable), and ADG No. 25 (Psychosocial: Recurrent or Persistent, Unstable).

^e^ Major physical ADGs refer to ADG No. 3 (Time Limited: Major), ADG No. 4 (Time Limited: Major–Primary Infection), ADG No. 9 (Likely to Recur: Progressive), ADG No. 11 (Chronic Medical: Unstable), ADG No. 16 (Chronic Specialty: Unstable–Orthopedic), ADG No. 22 (Injuries/Adverse Effects: Major), and ADG No. 32 (Malignant Neoplasm).

^f^ The percentage sum is more than 100% because a female may have had more than 1 teen pregnancy.

^g^ Of these 60 037 births, 59 485 (99.1%) were live births, 384 (0.6%) stillbirths, and 168 (0.3%) stillbirths and live births.

In the main model, there were 6030 premature deaths (crude rate, 1.9 per 10 000 person-years [95% CI, 1.9-2.0 per 10 000 person-years]) in the reference group without a teen pregnancy, increasing to 701 deaths (crude rate, 4.1 per 10 000 person-years [95% CI, 3.8-4.5 per 10 000 person-years]) among those with 1 teen pregnancy, and 345 deaths (crude rate, 6.1 per 10 000 person-years [95% CI, 5.5-6.8 per 10 000 person-years]) among those with at least 2 teen pregnancies (Table 2). Adjusted HRs (AHRs) were 1.51 (95% CI, 1.39-1.63) for those with 1 pregnancy and 2.14 (95% CI, 1.92-2.39) for those with 2 or more pregnancies.

**Table 2.** Risk of Premature Mortality From 12 Years of Age Onward, in Association With the Number of Teen Pregnancies a Female Had Between 12 and 19 Years of Age (Main Model)

Exposure: teen pregnancies at ages 12-19 y (No. [%])	No. of person-years of follow-up, median (IQR)	Outcome of premature mortality, starting at age 12 y		
		No. (incidence rate [95% CI] per 10 000 person-years)	Unadjusted HR (95% CI)	Adjusted HR (95% CI)^a^
0 (n = 2 079 805 [92.7])	13 (6-20)	6030 (1.9) [1.9-2.0]	1.00 [Reference]	1.00 [Reference]
1 (n = 121 276 [5.4])	19 (13-24)	701 (4.1) [3.8-4.5]	1.66 (1.54-1.80)	1.51 (1.39-1.63)
≥2 (n = 41 848 [1.9])	19 (14-23)	345 (6.1) [5.5-6.8]	2.46 (2.20-2.74)	2.14 (1.92-2.39)

Abbreviation: HR, hazard ratio.

^a^ Adjusted for each female’s year of birth, number of comorbidity Aggregated Diagnosis Groups at 9 to 11 years of age (≤2, 3-4, 5-6, or ≥7), area-level educational attainment less than high school (when teen was 12 years of age), time-varying residential income quintile at 12 to 19 years of age, and time-varying rural residence at 12 to 19 years of age.

In additional analysis 1, on resetting time zero to start at 20 years of age, the AHRs were largely unchanged (eTable 3 in Supplement 1). In additional analysis 2, further adjusting for time-varying mental health factors at 12 to 19 years of age, the AHRs were slightly more attenuated but remained significant after 1 teen pregnancy (1.31 [95% CI, 1.21-1.42]) or at least 2 teen pregnancies (1.78 [95% CI, 1.59-1.99]) (eTable 4 in Supplement 1). Relative to no teen pregnancy, the AHR for premature death was 1.41 (95% CI, 1.29-1.54) if the first teen pregnancy ended in an induced abortion and 2.10 (95% CI, 1.91-2.31) if it ended in a miscarriage or birth (Table 3).

**Table 3.** Risk of Premature Mortality From 12 Years of Age Onward, in Association With How the Teen Pregnancy Ended Between 12 and 19 Years of Age

Time-varying exposure of any teen pregnancy at ages 12-19 y^a^	Outcome of premature mortality, starting at age 12 y		
	No. (incidence rate [95% CI] per 10 000 person-years)	Unadjusted HR (95% CI)	Adjusted HR (95% CI)^b^
No teen pregnancy (n = 2 079 805)	6030 (1.9) [1.9-2.0]	1.00 [Reference]	1.00 [Reference]
Teen pregnancy resulting in an induced abortion (n = 98 759)	547 (3.8) [3.5-4.1]	1.45 (1.33-1.58)	1.41 (1.29-1.54)
Teen pregnancy resulting in a live birth, stillbirth, miscarriage, or ectopic pregnancy (n = 64 365)	499 (6.2) [5.7-6.8]	2.69 (2.45-2.95)	2.10 (1.91-2.31)

Abbreviation: HR, hazard ratio.

^a^ If more than 1 teen pregnancy occurred between 12 and 19 years of age, then the earliest one was considered.

^b^ Adjusted for each female’s year of birth, number of comorbidity Aggregated Diagnosis Groups at 9 to 11 years of age (≤2, 3-4, 5-6, or ≥7 years), area-level educational attainment less than high school (when teen was 12 years of age), time-varying residential income quintile at 12 to 19 years of age, and time-varying rural residence at 12 to 19 years of age.

Comparing those with vs those without a teen pregnancy, the AHR for premature death from a noninjury cause was significantly higher (1.25 [95% CI, 1.12-1.40]), and the AHRs for those from unintentional injury (2.06 [95% CI, 1.75-2.43]) and intentional injury (2.02 [95% CI, 1.54-2.65]) were even higher (Table 4). Among those with a teen pregnancy, noninjury-related premature mortality was more common (incidence rate, 2.0 per 10 000 person-years [95% CI, 1.8-2.2 per 10 000 person-years]) than either unintentional (incidence rate, 1.0 per 10 000 person-years [95% CI, 0.9-1.2 per 10 000 person-years]) or intentional (incidence rate, 0.4 per 10 000 person-years [95% CI, 0.3-0.5 per 10 000 person-years]) deaths from injury. Those who had a teen pregnancy before 16 years of age had the highest incidence rate of premature death and a corresponding AHR of 2.00 (95% CI, 1.68-2.39) (Table 5).

**Table 4.** Risk of Premature Mortality From 12 Years of Age Onward, in Association With Pregnancy Between 12 and 19 Years of Age, Further Specified by the Nature of the Death

Outcome	Time-varying exposure of teen pregnancy at ages 12-19 y	No. (incidence rate [95% CI] per 10 000 person-years)	Unadjusted HR (95% CI)	Adjusted HR (95% CI)^a^
Noninjury-related premature mortality	No (n = 1 907 106)	2864 (1.1) [1.1-1.2]	1.00 [Reference]	1.00 [Reference]
	Yes (n = 163 078)	354 (2.0) [1.8-2.2]	1.38 (1.23-1.54)	1.25 (1.12-1.40)
Injury-related premature mortality of an unintentional nature	No (n = 1 907 106)	976 (0.4) [0.4-0.4]	1.00 [Reference]	1.00 [Reference]
	Yes (n = 163 078)	184 (1.0) [0.9-1.2]	2.23 (1.89-2.62)	2.06 (1.75-2.43)
Injury-related premature mortality of an intentional nature^b^	No (n = 1 907 106)	376 (0.1) [0.1-0.2]	1.00 [Reference]	1.00 [Reference]
	Yes (n = 163 078)	67 (0.4) [0.3-0.5]	2.29 (1.75-2.99)	2.02 (1.54-2.65)

Abbreviation: HR, hazard ratio.

^a^ Adjusted for each female’s year of birth, number of comorbidity Aggregated Diagnosis Groups at 9 to 11 years of age (≤2, 3-4, 5-6, or ≥7 years), area-level educational attainment less than high school (when teen was 12 years of age), time-varying residential income quintile at 12 to 19 years of age, and time-varying rural residence at 12 to 19 years of age.

^b^ For women without a teen pregnancy, 761 of fatal intentional injuries (85.1%) were due to self-harm (*International Classification of Diseases, Ninth Revision* [*ICD-9*] codes E950-E959; *International Statistical Classification of Diseases and Related Health Problems, Tenth Revision, Enhanced for Canada* [*ICD-10CA*] codes X60-X84 and Y87.0), and 133 (14.9%) were due to assault (*ICD-9* codes E960-E969; *ICD-10CA* codes X85-Y09 and Y87.1). For females with a teen pregnancy, 134 of fatal intentional injuries (74.4%) were due to self-harm and 46 (25.6%) were due to assault. This analysis is limited to the assessment of deaths up to December 2018, the last available date for death certificate data.

**Table 5.** Risk of Premature Mortality From 12 Years of Age Onward, in Association With the Age at Which a Female Experienced Her First Pregnancy Between 12 and 19 Years of Age

Exposure: age at the first teen pregnancy^a^	Outcome of premature mortality, starting at age 12 y		
	No. (incidence rate [95% CI] per 10 000 person-years)	Unadjusted HR (95% CI)	Adjusted HR (95% CI)^b^
No teen pregnancy (n = 2 079 805)	6030 (1.9) [1.9-2.0]	1.00 [Reference]	1.00 [Reference]
12-15 y (n = 13 656)	125 (5.5) [4.6-6.6]	2.36 (1.98-2.82)	2.00 (1.68-2.39)
16-17 y (n = 56 034)	391 (4.8) [4.3-5.2]	1.95 (1.76-2.16)	1.74 (1.57-1.93)
18-19 y (n = 93 434)	530 (4.4) [4.0-4.8]	1.71 (1.56-1.87)	1.56 (1.42-1.70)

Abbreviation: HR, hazard ratio.

^a^ If more than 1 teen pregnancy arose between 12 and 19 years of age, then the earliest one was considered.

^b^ Adjusted for each female’s year of birth, number of comorbidity Aggregated Diagnosis Groups at 9 to 11 years of age (≤2, 3-4, 5-6, or ≥7), area-level educational attainment less than high school (when teen was 12 years of age), time-varying residential income quintile at 12 to 19 years of age, and time-varying rural residence at 12 to 19 years of age.

## Discussion

In this population-based cohort study of 2.2 million females followed up within a universal health care system, the risk of premature death by approximately 31 years of age was 1.5 times higher among those who had 1 teen pregnancy and 2.1 times higher among those with at least 2 teen pregnancies. The associated risk was higher if the teen pregnancy ended in a miscarriage or birth (ie, spontaneously) and for deaths due to injury. A teen pregnancy before 16 years of age had the highest associated risk of premature death.

### Strengths and Limitations

This study has some strengths. It was completed within a universal health care system that captures all teen pregnancies resulting in a hospital live birth, stillbirth, or ectopic pregnancy, as well as drug- and procedure-induced abortions and miscarriages managed within an inpatient or ambulatory setting.^16,17^

Prior studies of teen pregnancy and future outcomes were based largely on survey data typically administered in the fifth decade of life and, therefore, were prone to survivor bias.^10,11,12^ Published studies were also underpowered to detect deaths during adolescence and early adulthood, including subtypes of death by intentional vs unintentional injury. Prior studies were rarely completed within a universal health care system, such that teen pregnancies ending in drug-induced abortion could not be completely ascertained nor with sufficient follow-up into adulthood.

This study also has some limitations. We could not identify gender in our administrative data sets, only biological sex at birth. We also did not explore race or ethnicity as a confounder of the association between teen pregnancy and premature mortality for several reasons. First, we did not possess a data source that accurately identifies race and ethnicity, especially among Canadian-born women. Second, we were unaware of the reasons why race and ethnicity would be associated with teen pregnancy or premature mortality beyond the known higher rates of economic disadvantage and structural racism experienced by certain racial and ethnic groups^20^ and the ensuing health risks to an affected family,^21^ including a higher likelihood of ACEs^22^ and intergenerational teen pregnancies.^16^ Adversity during childhood is associated with a greater likelihood of teen pregnancy^10^ and for developing a broad array of chronic physical and psychiatric disorders.^23^ Even so, future strategies aimed at reducing ACEs, teen pregnancies, or the risks of premature death should carefully consider race and ethnicity within the context of exposure to economic or social disadvantage.

In the present study, the overall large number of fatalities enabled the generation of precise risk estimates, including deaths associated with intentional and unintentional injury or mortality.^4^ Absolute risk differences could not be calculated from the Cox proportional hazards regression models given the time time-varying nature of the exposure.^24^ Although women with a teen pregnancy had a higher rate of self-harm history between 12 and 19 years of age than those without a teen pregnancy, we did not evaluate interpersonal violence, substance use,^25^ or psychiatric illness.^11^ For example, in a French population-based cohort study comparing pregnant adolescents aged 12 to 18 years with age-matched nonpregnant adolescents, the HR for subsequent hospitalization for nonlethal self-harm was 3.1 (95% CI, 2.6-3.7).^18^ Hence, assessment of these and other prevalent outcomes should provide a powerful examination of important antecedent conditions that can compromise physical health and life expectancy.^26^

### Implications for Policy and Clinical Practice

Evidence suggests that higher ACE scores in early childhood are a major factor associated with premature death during adolescence and midadulthood.^9^ Although there is a need to identify and reduce factors associated with ACEs,^14,15,22,27^ including family instability, poverty, crowded housing, and parental separation, these factors have typically been evaluated in research studies up to the preteen years.^9^ Because individuals who have a history of ACEs are also more likely to engage in sexual risk taking and to experience teenage pregnancy,^27^ a teen pregnancy may be another time point for identifying some individuals at greater risk of premature mortality and morbidity to facilitate access to appropriate supports. Such individuals may need counseling and assistance in dealing with the lingering trauma of ACEs, access to positive role models, supports to remain in school, and opportunities to promote self-efficacy during and after their teen years.^28,29^

The present study included all recognized teen pregnancies in Ontario, as well as how the pregnancy ended. Deaths as a direct consequence of a procedural or pharmaceutical abortion are extremely rare.^17^ In a cohort study from France, the risk of hospitalization for nonlethal self-harm among teenagers was highest after induced abortion (HR, 3.5 [95% CI, 2.9-4.2]).^18^ In the present study, teenagers whose pregnancy ended in an induced abortion were at somewhat higher risk of premature mortality, whereas the risk was even higher for those with a pregnancy that ended spontaneously in a birth or miscarriage. Together, the age at first teen pregnancy, the cumulative number of pregnancies, and the outcome of a teen pregnancy might each inform the targeting of strategies for the prevention of premature mortality among females.

## Conclusions

This cohort study suggests that teen pregnancy may be a readily identifiable marker for subsequent risk of premature mortality in early adulthood. Apparent protective factors for the prevention of adolescent pregnancy include a stable family, school and peer support, open communication with adult mentors or parents about contraception use, free access to contraception, and female empowerment to abstain from unwanted or unplanned intercourse.^16,30^ Some of the former factors, among others, may also reduce the risk of youth suicide and self-harm.^31^ It remains to be determined whether there is additive value in including teenage pregnancy in the prevention of premature mortality among young and middle-aged women.

## Notes

Supplement 1. **eTable 1.** List of ICES Databases Used in the Current Study **eTable 2.** Diagnostic and Procedural Codes Used to Identify the Cohort, Comorbidity and Outcome Variables **eTable 3.** (*Additional Analysis 1*, as a Modification of the Main Model) **eTable 4.** (*Additional Analysis 2*, as a Modification of the Main Model)

Supplement 2.
Data Sharing Statement
