# Supplementary material for: Teen Pregnancy and Risk of Premature Mortality
Source: JAMA Netw Open. 2024 Mar 14;7(3):e241833. doi: 10.1001/jamanetworkopen.2024.1833 (PMC10940968; doi:10.1001/jamanetworkopen.2024.1833)
Supplement: Supplement 1. — eTable 1. List of ICES Databases Used in the Current Study eTable 2. Diagnostic and Procedural Codes Used to Identify the Cohort, Comorbidity and Outcome Variables eTable 3. (Additional Analysis 1, as a Modification of the Main Model) eTable 4. (Additional Analysis 2, as a Modification of the Main Model) [file jamanetwopen-e241833-s001.pdf]

# Supplementary Online Content

Ray JG, Fu L, Austin PC, et al. Teen pregnancy and risk of premature mortality.
*JAMA Netw Open.* 2024;7(3):e241833. doi:10.1001/jamanetworkopen.2024.1833

**eTable 1.** List of ICES Databases Used in the Current Study
**eTable 2.** Diagnostic and Procedural Codes Used to Identify the Cohort, Comorbidity and Outcome Variables
**eTable 3.** (*Additional Analysis 1*, as a Modification of the Main Model)
**eTable 4.** (*Additional Analysis 2*, as a Modification of the Main Model)

This supplementary material has been provided by the authors to give readers additional information about their work.

© 2024 Ray JG et al. *JAMA Network Open.*

**eTable 1.** List of ICES Databases Used in the Current Study

| **Dataset name** | **Description** |
|---|---|
| Canadian Institute for Health Information Discharge Abstract Database (CIHI-DAD) | Captures all in-patient hospital admission records including obstetric deliveries and deaths. Diagnostic codes are based on the *International Statistical Classification of Diseases and Related Health Problems, Tenth Revision, Canada* (*ICD-10-CA*), and procedural codes are based on the *Canadian Classification of Health Interventions (CCI)*. |
| Linked Delivering Mothers and Newborns (MOMBABY) | Derived from CIHI-DAD, provides linked inpatient hospital admission records of mothers and their infants. |
| Registered Persons Database (RPDB) | Includes vital status and sociodemographic information about all individuals who have ever received an Ontario Health Insurance Plan (OHIP) number (e.g., date of birth, sex, and postal code). |
| Postal Code Conversion File Plus (PCCF+) | A digital file that links the Canada Post Corporation (CPC) six-character postal code and Statistics Canada's standard geographic areas (e.g., dissemination area). Area-level income quintiles ranges from Q1 (lowest) to Q5 (highest) income neighbourhoods. |
| Statistic Canada Census | Information from the Canadian Census, statistical information about the population including population counts and various levels of geography (e.g., census metropolitan areas, communities, census tracts etc.) |
| Office of the Registrar General - Deaths (ORG-D) | An annual dataset including the date and cause of death, for all deaths registered in Ontario |
| Aggregated Diagnosis Groups (ADG)® | The Johns Hopkins Adjusted Clinical Groups (ACGs)® system assigns each ICD codes to one of 32 diagnosis clusters known as Aggregated Diagnosis Groups (ADG). Individual diseases or conditions are placed into a single ADG based on five clinical dimensions: duration of the condition; severity of the condition; diagnostic certainty; etiology of the condition; and specialty care involvement. ICD codes within the same ADG are similar in both clinical criteria and expected need for healthcare resource. Each individual may have diagnoses belonging to between zero and 32 ADGs. |
| Ontario Drug Benefit Claims database | Information (recipients, payments, claims, practitioners) for the Ontario Drug Benefit Program. Drug identification number is provided by IQVIA Solutions Canada Inc. These databases are used in the creation of ICES-derived cohorts. |

© 2024 Ray JG et al. *JAMA Network Open.*

**eTable 2.** Diagnostic and Procedural Codes Used to Identify the Cohort, Comorbidity and Outcome Variables

| Assessment | Procedure, disease or demographic | Discharge Abstract Database (DAD) and National Ambulatory Care Reporting System (NACRS): CCP or CCI procedure codes or ICD-9 or ICD-10-CA diagnosis codes | Ontario Health Insurance Plan (OHIP) Database: Professional Fee Code or ICD-9 diagnostic code | Other source |
|---|---|---|---|---|
| ***Cohort inclusion criteria*** | Females, aged 12 years between 1991 and 2021, with OHIP eligibility at start of age 12 years | -- | -- | RPDB |
| ***Exclusion criteria*** | Loss of OHIP eligibility any time before 12th birthday | -- | -- | RPDB |
| ***Main exposure*** | Time varying number (0, 1, 2, or 3+) of teen pregnancies between ages 12 to 19 completed years, with a pregnancy defined as the recorded date of a livebirth, stillbirth, induced abortion or miscarriage, as follows: | | | |
| | a) Livebirth from 20 weeks' gestation to 42 weeks' gestation | Main patient service code indicating "obstetrical delivery"<br>(MOMBABY - includes linked DAD inpatient admission records of delivering mothers and their newborns) | -- | MOMBABY (https://datadictionary.ices . on.ca/Applications/DataDictionary/Library.aspx?Library=MOMBABY)<br>m_stillbirth='F' |
| | b) Stillbirth from 20 weeks' gestation to 42 weeks' gestation | -- | -- | MOMBABY<br>m_stillbirth='T' |
| | c) Miscarriage or ectopic pregnancy < 20 weeks | ICD-9 632, 633, 634<br>ICD-10-CA O00, O021, O03 | -- | Fee code: A920, P001 AND ICD-9: 632, 633, 634, 640;<br>OR Fee code: A922;<br>OR Fee code: S752, S785 AND ICD-9: 632, 633, 634, 640; |

© 2024 Ray JG et al. *JAMA Network Open.*

| | | | | |
|---|---|---|---|---|
| | d) Induced abortion at any gestational age | *Procedural induced abortion:*<br>CCP: 81.01, 87.0, 87.1, 87.21, 87.29<br>and ICD-9: 635<br><br>CCI: 5CA89, 5CA88, 5CA20FK, 5CA24<br>and ICD-10-CA: O04, O08<br><br>*Pharmacuetical induced abortion with mifepristone-misoprostol:* | Fee Code: S752, S785, A920, P001 and ICD-9: 635, 895<br><br><br>--<br><br>-- | OR Fee code: S756, S768, S784, S770<br><br><br>--<br><br>Drug Identification Number: 02444038 in Ontario Drug Benefit Claims database |
| ***Secondary exposures*** | Nature of any teen pregnancy between ages 12-19 years:<br>• No teen pregnancy<br>• Teen pregnancy ending in a livebirth or stillbirth or miscarriage or ectopic pregnancy<br>• Teen pregnancy ending in an induced abortion<br><br>NB: If more than one teen pregnancy between ages 12-19 y, then chose the earliest one | Same as above | Same as above | Same as above |
| | Age at the first teen pregnancy:<br>• No teen pregnancy<br>• 12-13 y<br>• 14-15 y<br>• 16-17 y<br>• 18-19 y<br><br>NB: If more than one teen pregnancy between ages 12-19 y, then chose the earliest one. | Same as above | Same as above | Same as above |

© 2024 Ray JG et al. *JAMA Network Open.*

| | | | | |
|---|---|---|---|---|
| ***Main study outcome*** | All-cause mortality, starting at age 12 y | -- | -- | RPDB: DTHDATE |
| ***Secondary study outcomes*** | Nature of death, starting at age 12 years (for available deaths up to December 2018):<br>• Non-injury related premature mortality<br>• Injury-related premature mortality of a non-intentional nature<br>• Injury-related premature mortality of an intentional nature<br>NB: If more than one teen pregnancy between ages 12-19 y, then chose the earliest one | Death not related to injury, where injury is defined immediately below.<br>NACRS or DAD:<br>ICD-9 E800-E929 (excluding E870-E879)<br>ICD-10-CA V01-X59, Y85-Y86<br>NACRS or DAD:<br>ICD-9 E950-E959, E960-E969<br>ICD-10-CA X60-Y09, Y87.0, Y87.1 | --<br>--<br>-- | ORGD: cod_underlying_icd10, up to December 2018<br>ORGD: cod_underlying_icd10, up to December 2018<br>ORGD: cod_underlying_icd10, up to December 2018 |
| | All-cause mortality, starting at age 20 y (Additional analysis as a modification of the main model). NB: This model only considers women alive at their 20th birthday, and who had continuous OHIP eligibility any time before their 12th birthday, and up to their 20$^{th}$ birthday | -- | -- | RPDB |
| ***Covariates or baseline variables*** | Year of birth of the woman | -- | -- | RPDB |
| | History of self-harm or overdose from before age 12 years | ICD-9 E950-E959<br>ICD-10-CA X60-X84, Y87.0 | -- | -- |

© 2024 Ray JG et al. *JAMA Network Open.*

| | | | | |
|---|---|---|---|---|
| | Number of comorbidity ADGs at ages 9-11 years (0 to 2, 3 to 4, 5 to 6 or ≥ 7) | -- | -- | ADGs are obtained from diagnosis codes in DAD, SDS and NACRS using The Johns Hopkins ACG® System software |
| | Area-level education attainment less than high school (when teen was age 12 years), based on 6-digit postal code | -- | -- | Statistics Canada census, defined as the percentage of the local population aged 25 to 64 years with no high school certificate, diploma or degree |
| | Time-varying residential income quintile starting at ages 12-19 years, based on 6-digit postal code | -- | -- | The Postal Code Conversion File Plus is a digital file that links the Canada Post Corporation six-character postal code and Statistics Canada's standard geographic areas (e.g., dissemination area). Area-level income quintiles ranges from Q1 (lowest) to Q5 (highest) income neighbourhoods. See "Box 2" at https://shorturl.at/befAS |
| | Time-varying rural residency at ages 12-19 years, based on 6-digit postal code | -- | -- | Statistics Canada census |
| ***Censoring variables*** | Death as the outcome | -- | -- | RPDB |
| | End of study follow-up | -- | -- | RPDB |
| | Date of last contact in administrative datasets | -- | -- | RPDB |
| ***Additional analysis 1*** | Only include women alive at their 20th birthday, who had continuous OHIP eligibility between ages 12-19 years. The exposure is the total number of teen pregnancies between ages 12-19 completed y. The | -- | -- | -- |

© 2024 Ray JG et al. *JAMA Network Open.*

| | | | | |
|---|---|---|---|---|
| | outcome is all-cause premature mortality, starting at a woman's 20$^{th}$ birthday | | | |
| ***Additional analysis 2*** | Further adding to the main model and time-varying Mental Health ADG #23 (Psychosocial: Time Limited, Minor), #24 (Psychosocial: Recurrent or Persistent, Stable) and #25 (Psychosocial: Recurrent or Persistent, Unstable) at ages 12-19 y | | | |

CCP Canadian Classification of Diagnostic, Therapeutic and Surgical Procedures (CCP) codes
CCI Canadian Classification of Health Interventions (CCI) codes
ICD-10-CA International Classification of Diseases, 10th Revision, Canada; OHIP Ontario Health Insurance Plan.
ICD-9 International Classification of Diseases, 9th Revision.
ADG Aggregated Diagnosis Group
NACRS National Ambulatory Care Reporting System
OHIP Ontario Health Insurance Plan
RPDB Registered Persons Database
SDS Same Day Surgery Database

© 2024 Ray JG et al. *JAMA Network Open.*

**eTable 3.** (*Additional Analysis 1*, as a Modification of the Main Model). Risk of the Secondary Outcome of Premature Mortality From Age 20 Years Onward, in Association With the Number of Teen Pregnancies a Woman Had Between Ages 12 to 19 y. This model only considers women who were alive at their 20th birthday.

| **Exposure** | | **Outcome of premature mortality, starting at age 20 y** | | |
|---|---|---|---|---|
| **Total number of teen pregnancies from ages 12 to 19 y** | **Median (IQR) number of person-years of follow-up** | **No. (incidence rate [95% CI] per 10 000 person-years)** | **Unadjusted hazard ratio (95% CI)** | **Adjusted hazard ratio (95% CI) [a]** |
| *0 (N = 1,480,722)* | 9 (4-15) | 4099 (2.6, 2.5-2.7) | 1.00 (referent) | 1.00 (referent) |
| *1 (N = 113,362)* | 11 (6-16) | 626 (4.5, 4.2-4.9) | 1.67 (1.54-1.82) | 1.53 (1.41-1.67) |
| *≥ 2 (N = 40,051)* | 11 (7-16) | 326 (6.7, 6.0-7.5) | 2.54 (2.27-2.85) | 2.22 (1.98-2.49) |

[a]Adjusted for each woman's year of birth, number of comorbidity Aggregated Diagnosis Groups at ages 9-11 y (0 to 2, 3 to 4, 5 to 6 or ≥ 7), area-level education attainment less than high school (when teen was age 20 y), income quintile at age 20 y, and rural residence at age 20 y.

© 2024 Ray JG et al. *JAMA Network Open.*

**eTable 4.** (*Additional Analysis 2*, as a Modification of the Main Model). Risk of Premature Mortality From Age 12 Years Onward, in Association With the Number of Teen Pregnancies a Woman Had Between Ages 12 to 19 y. This model further adjusts for time-varying mental health factors at ages 12 to 19 years.

| **Exposure** | | **Outcome of premature mortality, starting at age 12 y** | | |
|---|---|---|---|---|
| **Number of teen pregnancies from ages 12-19 y (N [%])** | **Median (IQR) number of person-years of follow-up** | **No. (incidence rate [95% CI] per 10 000 person-years)** | **Unadjusted hazard ratio (95% CI)** | **Adjusted hazard ratio (95% CI)** [a] |
| *0 (N = 2,079,805 [92.7])* | 13 (6, 20) | 6030 (1.9, 1.9-2.0) | 1.00 (referent) | 1.00 (referent) |
| *1 (N = 121,276 [5.4])* | 19 (13, 24) | 701 (4.1, 3.8-4.5) | 1.66 (1.54-1.80) | 1.31 (1.21-1.42) |
| *≥ 2 (N = 41,848 [1.9])* | 19 (14, 23) | 345 (6.1, 5.5-6.8) | 2.46 (2.20-2.74) | 1.78 (1.59-1.99) |

[a] Adjusted for each woman's year of birth, number of comorbidity Aggregated Diagnosis Groups at ages 9-11 y (0 to 2, 3 to 4, 5 to 6 or ≥ 7), area-level education attainment less than high school (when teen was age 12 y), time-varying residential income quintile at ages 12-19 y, time-varying rural residence at age 12-19 y, and time-varying Mental Health Aggregated Diagnosis Groups #23 (Psychosocial: Time Limited, Minor), #24 (Psychosocial: Recurrent or Persistent, Stable) and #25 (Psychosocial: Recurrent or Persistent, Unstable) at ages 12-19 y.

© 2024 Ray JG et al. *JAMA Network Open.*
