# Supplementary material for: Teen Pregnancy and Risk of Premature Mortality
Source: JAMA Netw Open. 2024 Mar 14;7(3):e241833. doi: 10.1001/jamanetworkopen.2024.1833 (PMC10940968; doi:10.1001/jamanetworkopen.2024.1833)
Supplement: Supplement 2. — Data Sharing Statement [file jamanetwopen-e241833-s002.pdf]

Data Sharing Statement

Ray. Teen Pregnancy and Risk of Premature Mortality. *JAMA Netw Open*. Published March 14, 2024. doi:10.1001/jamanetworkopen.2024.1833

Data
Data available: No
